# Profile Optimisation of a Solid Modular Hob in the Machining of Gears Made of Classic and Unusual, Innovative Materials

**DOI:** 10.3390/ma17092049

**Published:** 2024-04-26

**Authors:** Andrzej Piotrowski, Artur Tyliszczak

**Affiliations:** 1 Department of Technology and Automation, Czestochowa University of Technology, 42-201 Czestochowa, Poland; andrzej.piotrowski@pcz.pl; 2 Department of Thermal Machinery, Czestochowa University of Technology, 42-201 Czestochowa, Poland

**Keywords:** modular solid hob, mathematical model of the tool, improving the accuracy of the hob, numerical simulation

## Abstract

Modular hobs are tools with very complex geometry. Regardless of the material of the gear wheels, they determine the accuracy of the gears made in the hobbing machining process. Gears are made of various, often innovative materials depending on the requirements. Sometimes, the materials are characterised by very high hardness (over 65 HRC). The mathematical basis for describing the faces of a hob presented in the article allows for modifying the rack profile shaping the gear wheel’s teeth. The model’s universality makes it possible to perform numerical simulations of the influence of individual parameters of the hob creation process (geometry of the grinding wheels and their setting in the shaping process) on the profile of the rake and flank surfaces. The cutting edge (rack edge) is the locus of points belonging to both of these surfaces and thus directly impacts the accuracy of the gear wheel that is shaped in the hobbing process. The article summarises the authors’ long-term cooperation with the industry, resulting in a series of articles devoted to hobs. The issues presented in the article are significant to the machinery industry and hob manufacturers.

## 1. Introduction

When metallurgists, chemists, and industrialists develop new, innovative materials, they believe they can be a panacea for the chosen technological problem. It fulfils all the criteria set by users in terms of strength, quietness, durability, and accuracy. However, industrial practice has shown that this is not entirely true. Achieving the required accuracy in gear hobbing with geometrically complex tools is particularly difficult. The gears were produced in low-accuracy classes and required further grinding or lapping. The result was increased costs and longer production times. This was the basis for establishing cooperation with the Polish machinery industry and enabling research to be carried out to analyse the impact of material, machining method, and tool geometry on the accuracy of the gears. The research topic was derived directly from industry demand. In Polish conditions, hobs were produced in classes C and B. The “trial and error” method was employed to enhance their precision, which was time-consuming and did not always result in the desired outcome of a class A or AA milling cutter.

Modular hobs are the primary tool for milling involute cylindrical gears with straight and helical teeth. The accuracy of the hob determines the accuracy of the gear. By standards and industrial practice, they enable the production of gears in classes from 10 (class C hobs) to 5 (Class AAA hobs), and even Class 4 (Class AAAA hobs) [1,2,3,4,5,6,7,8]. The higher the hob class, the higher the price of the tool. For instance, a Class AAA hob is many times more expensive than a Class B or C hob. However, this translates into the machining of gears. Gears machined with a Class AAA or AAAA hob need not be ground and lapped. This is very important in the automotive industry, where we strive to reduce production times as much as possible and simplify the gear manufacturing process. In response to this demand, our objective is to enhance the precision of hobs. This is not feasible without the application of mathematical modelling and computer simulation.

**Figure 1:**
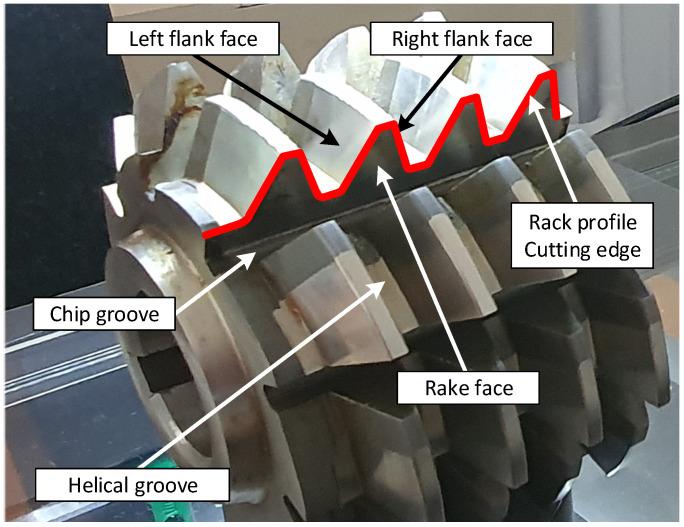
Classic solid modular hob—the main parts.

Hobs enable the production of palloid bevel gears, worm wheels, worms, splines, chain wheels, ratchets, and many others [5,9]. The hobs are used in various industries to process a variety of materials, from soft and easily machinable polymer materials to high-hardness steels or extremely hard innovative materials. They can be generally divided into two primary groups: solid modular hobs (Figure 1) and composite hobs with inserts made of cemented carbide, often with parts made of diamond or borazon [10]. Solid hobs are characterised by a much higher accuracy than composite tools.

The latest gear manufacturing technology is based on additive manufacturing technology. This has been the subject of numerous research and scientific studies [11]. Here, we can distinguish between different types of 3D printing. In the case of metal-based materials, laser sintering, as described in the work of Jardin, Tuninetti, Tchuindjang, Hashemi, Carrus, and Pinkerton [12], is of great importance. Various powdered materials are employed, including innovative ones that fuse together when exposed to high temperatures. The advantage of this method is that any profile shape or modification of the gear tooth can be achieved. However, the disadvantage of sintering is that it is extremely time-consuming (the printing process takes up to several tens of hours) and costly. This method is well suited to the creation of single experimental parts and the regenerating of parts, as demonstrated by Graf, Ammer, Gumenyuk, and Rethmeier [13]; and Pinkerton, Wang, and Li [14]. However, the accuracy requirements for gears render this method unsuitable. The workpiece requires further finishing after sintering. In mass production, the dominant process is still machining, which involves cutting using hobs.

Gears are the basis for constructing the tooth gears used in the precision and automotive industries and for constructing heavy tooth gears in the steel industry [15]. Their quality translates into durability and the quiet operation of tooth gears. They are made on special hobbing machines [16]. Depending on the application of gears, various materials are used for gears, from classic ones (steels) to innovative materials for special applications (superhard materials or specialised polymer materials). According to the theory cited by many authors [1,5,6,9,17], the gear tooth has the shape of an involute and is created by rolling a straight line in a circle without slipping. Accordingly, the lateral profile of the rack tooth must be straight, as was explained by Dzierżkowski [1] and Kunstetter [9]. This means that, regardless of the material selected by the manufacturer, the accuracy of the hob, and more precisely, the cutting-edge profile (rack profile) (Figure 1), has a decisive impact on the accuracy of the gear wheel machined with it. This is a universal relationship. A hypothesis must, therefore, be formulated. Since the hob determines the accuracy of the gear, it is necessary to improve its accuracy as much as possible. The tooth profile is influenced by the flank and rake surfaces, which determine the hob’s accuracy. A mathematical model should be created to describe the tool geometry. The model can then be employed to analyse and achieve the primary objective of enhancing the precision of the hob.

Solid modular hobs have been known for almost 200 years. The most important stages in the development of their design were the work of J. Ramseden from 1777, the patent of J. Whitworth from 1835, the work of Pfaff from 1839, the patent of R. Whitehead from 1835, and the work of Ch. Schiele from 1855 [18]. Over this long period, their design has been subject to many changes and modernisation. Despite the rapid development of composite tools with cemented carbide inserts, solid hobs are still produced, developed [3,4,19,20,21,22], and used in factories, particularly in automotive. This especially applies to tools with very high accuracy, i.e., Class AA to AAA and even Class AAAA hobs [8]. The analysis of hobs available on the market showed that high-speed steel is commonly used in the construction of modular hobs, particularly for cutting polymer materials, wood, and other materials with good machining properties [2,3,4,20,21,22]. On the other hand, various types of cemented carbides are used for hard machining difficult-to-cut or innovative materials characterised by admixtures of very hard alloying elements [2,3,4,10]. The faces of solid modular hobs are often covered with various layers that improve cutting properties and, in particular, facilitate chip flow. In the case of composite tools, the tool body is made of tool steel or high-speed steel, and the cutting inserts, inserted into specially designed sockets, are made of cemented carbide, ceramic materials, or inserts with borazon or diamond parts. An analysis of the literature and tool catalogues [2,3,4,10] showed that modern solid hobs are characterised by very high accuracy, the economy of high-speed steel, and improved cutting parameters. The technological process of producing hobs is complicated and requires several dozen operations [20,21,22]. Generally, during roughing and semi-finishing, helical grooves (Figure 1) are made on the circumference of the hob, most often on lathes. Chip grooves that form the rake faces are made on milling machines. In the next stage, tool staggering is performed. The accuracy of the hob is determined by the last operation, i.e., finishing machining carried out on specialised grinding machines [23]. According to many articles [24,25,26,27], this is a time-consuming and very expensive process, but it is decisive for the quality of the hob and the gear wheel made with it.

## 2. Mathematical Model of the Hob

Hobs are rotary tools with a repeating cutting profile around the circumference (Figure 1). The cutter rack consists of several to several dozen teeth, each of which has two flank faces (left and right) and a rake face. The tooth profile of the gear wheel is formed by the hob cutting edge created by the intersection of the flank faces with the rake face. In the case of an involute screw surface, the side cutting edges have a rectilinear shape in the cross-section tangential to the base cylinder. This is because, during gear milling, the hob forms a technological gear of the worm-gear type with the machined gear wheel. Straight cutting edges shape the involute profile of the gear tooth. This was proposed in 1856 by Ch. Schiele, who developed the hobbing method [1,18,24]. The term “hobbing” generally includes any machining where the object’s surface is the envelope of subsequent positions of the surface associated with the tool, which was the subject of many works by Koć [28,29]. However, this concept can be narrowed down, and it can be assumed that enveloping machining occurs only in the case of technological gears where the tool and the object are toothed elements.

Many works aimed at improving the accuracy of hobs include a geometric analysis that considers their technology. It was described by many authors, including Vasilis, Nectarios, and Aristomenis [30]; Dimitriou and Antoniadis [31], who focused on the CAD-based method to describe the kinematics in gear hobbing; Claudin and Rech [32]; Bouzakis and Lili, who presented a critical synthesis of analysis methods [33]; and Hsu and Fong [34]. The authors presented a detailed account of hobbing machining and the various mathematical models developed to optimise this process. However, Lechowski conducted the most significant and most beneficial studies in terms of computer modelling in this field [35]. However, while in the works of Macioszczyk [27], Lewkowicz [26,36], and Lechowski [35], the cases of shaping the side surface of the hobs with disc-type, finger-type, or cup-type wheels were treated as separate design tasks, in the works of Piotrowski and Nieszporek [24,37,38] a generalised algorithm covering all the above cases was presented.

**Figure 2:**
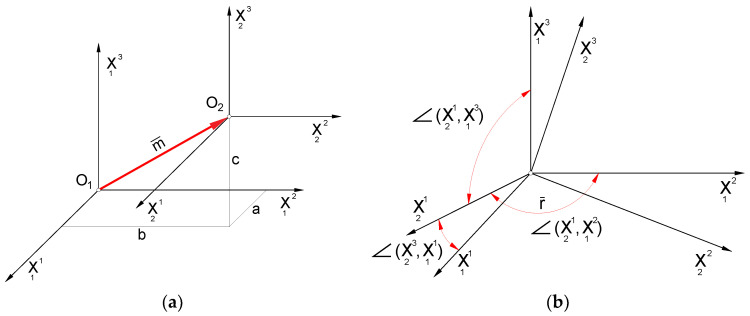
Division of movements: (**a**) translation and (**b**) rotation relative to the origin of the coordinate system.

A universal mathematical model of hob was created to describe the geometry and technological dependencies. The matrix calculus was used to describe subsequent transformations of the position of points defining the cutting edge (rack profile). This method is characterised by the separation of the transition between coordinate systems into translation (Figure 2a) and rotation (Figure 2b) and the description of rotations with third-degree matrices, which was proposed by Piotrowski [24], Nieszporek [37,38], Lechowski [35], and Koć [28,29]. This is of great practical importance, facilitating the use of this method in numerical science (computer calculations). The technique was also used by Koć [39] and then by Mazurkiewicz and Raczyk in their dissertations [40].

A parallel shift of the Ortho Cartesian coordinate system is called a transformation, which leads to the old coordinate system, O_1_, X_1_, Y_1_, and Z_1_ (Figure 2a), becoming the new coordinate system, O_2_, X_2_, Y_2_, and Z_2_, written as follows:

(1) $$\begin{array}{ccc} \left[\begin{array}{c} \underset{2}{X^{1}}\\ \underset{2}{X^{2}}\\ \underset{2}{X^{3}} \end{array}\right] & \begin{array}{cc} \begin{array}{cc} = & \left[\begin{array}{c} \underset{1}{X^{1}}\\ \underset{1}{X^{2}}\\ \underset{1}{X^{3}} \end{array}\right] \end{array} & - \end{array} & \left[\begin{array}{c} a\\ b\\ c \end{array}\right] \end{array}$$

where a, b, and c are the coordinates of the origin of the new system,$O_{2}$, in the $O_{1},X_{1}^{1},X_{1}^{2},X_{1}^{2}$ coordinate system.

When going from a $O_{2},X_{2}^{1},X_{2}^{2},X_{2}^{2}$ system to an inverse system $O_{1},X_{1}^{1},X_{1}^{2},X_{1}^{2}$, Formula (1) can be formally transformed, or the system $O_{1},X_{1}^{1},X_{1}^{2},X_{1}^{2}$ can be treated as a new system with coordinates of the point $O_{1}\left(-a,-b,-c\right)$.

A rotation of the Cartesian coordinate system (Figure 2b) is a transformation leading from the old coordinate system $O_{1},X_{1}^{1},X_{1}^{2},X_{1}^{2}$ to the new coordinate system $O_{2},X_{2}^{1},X_{2}^{2},X_{2}^{2}$, described as follows:

(2) $$\begin{array}{cccc} \left[\begin{array}{c} \underset{2}{X^{1}}\\ \underset{2}{X^{2}}\\ \underset{2}{X^{3}} \end{array}\right] & = & \left[\begin{array}{c} \begin{array}{ccc} \overset{I}{\overset{︷}{\cos(\underset{1}{X^{1}},\underset{2}{X^{1}})}} & \overset{II}{\overset{︷}{\cos(\underset{1}{X^{2}},\underset{2}{X^{1}})}} & \overset{III}{\overset{︷}{\cos(\underset{3}{X^{1}},\underset{2}{X^{1}})}} \end{array}\\ \begin{array}{ccc} \cos(\underset{1}{X^{1}},\underset{2}{X^{2}}) & \cos(\underset{1}{X^{2}},\underset{2}{X^{2}}) & \cos(\underset{3}{X^{1}},\underset{2}{X^{2}}) \end{array}\\ \underset{\left[MP\right]}{\underset{︸}{\begin{array}{ccc} \cos(\underset{1}{X^{1}},\underset{2}{X^{3}}) & \cos(\underset{1}{X^{2}},\underset{2}{X^{3}}) & \cos(\underset{3}{X^{1}},\underset{2}{X^{3}}) \end{array}}} \end{array}\right] & \left[\begin{array}{c} \underset{1}{X^{1}}\\ \underset{1}{X^{2}}\\ \underset{3}{X^{1}} \end{array}\right] \end{array}$$

It should be noted that, in the [MP] matrix (2), the axes of the old system always appear in columns I, II, and III, i.e., X^1^ in column I, the axis X^2^ in column II, and the axis X^3^ in column III. However, the rows contain the axes of the new system. The rotation matrix around the axes passing through the origin of the coordinate system can be written in general terms by the equation which was proposed by Nieszporek [38] and used by Piotrowski [24]:

(3) $$\begin{array}{ccc} \left[\bar{n},\varphi \right] & = & \left[\begin{array}{ccc} n_{1}^{2}+\left(1-n_{1}^{2}\right)\cos\varphi & n_{1}n_{2}\left(1-\cos\varphi \right)+n_{3}\sin\varphi & n_{1}n_{3}\left(1-\cos\varphi \right)-n_{2}\sin\varphi \\ n_{1}n_{2}\left(1-\cos\varphi \right)-n_{3}\sin\varphi & n_{2}^{2}+\left(1-n_{2}^{2}\right)\cos\varphi & n_{2}n_{3}\left(1-\cos\varphi \right)+n_{1}\sin\varphi \\ n_{1}n_{2}\left(1-\cos\varphi \right)+n_{2}\sin\varphi & n_{2}n_{3}\left(1-\cos\varphi \right)-n_{1}\sin\varphi & n_{3}^{2}+\left(1-n_{3}^{2}\right)\cos\varphi \end{array}\right] \end{array}$$

where $\bar{n}$—rotation axis versor; n_1_, n_2_, and n_3_—coordinates of the rotation axis vector in the Cartesian system; and φ—rotation angle.

To facilitate writing in a way that allows use in the programme, Formula (3) was written as follows:

(4) $$\begin{array}{c} n_{kk}=\cos\varphi +\left(1-\cos\varphi \right)n_{k}^{2}\\ n_{ij}=n_{i}n_{j}\left(1-\cos\varphi \right)+\varepsilon_{ijk}n_{k}\sin\varphi \end{array}$$

where i = j; k = j; ε_ijk_—Levi-Civita symbol; ε_ijk_ = ±1—depending on whether the permutation (ijk) is even or odd; i, j, k = 1, 2, or 3; and n_ij_ and n_kk_—matrix elements (3).

When rotating coordinate systems, it is necessary to define the direction of rotation about any axis of the coordinate system, i.e., to define the positive and negative direction of rotation. This has been performed in various ways in the literature, e.g., by Lechowski and Lewkowicz [36]. In the presented article, the positive direction of rotation is assumed to be the rotation from the known coordinate system to the searched coordinate system in a counterclockwise direction, viewed from the axis side. This assumption allows us to simplify the rotation matrix to the following form:

(5) $$\left[n,\varphi \right]$$

where n—number or vector of the rotation axis; and φ—rotation angle.

The simplest transformation resulting from writing the matrix in the form of (3)–(5) is the operation of transposing coordinate systems in the negative direction:

(6) $$\left[\bar{n},-\varphi \right]={\left[\bar{n},\varphi \right]}^{T}={\left[\bar{n},\varphi \right]}^{-1}$$

Successive rotations around the same axis are added up, which can be written down:

(7) $$\begin{array}{cccc} \left[\bar{n},\varphi_{1}\right] & \left[\bar{n},\varphi_{2}\right] & \ldots & \left[\bar{n},\varphi_{n}\right] \end{array}\begin{array}{cc} = & \left[\bar{n},\varphi_{1}+\varphi_{2}+\ldots +\varphi_{n}\right] \end{array}$$

where φ_1_, φ_2_, and φ_n_—angles of subsequent rotations around the axis in the $\bar{n}$ verso direction.

The envelope condition of the surface family using matrix calculus and the assumptions made above leads to the differentiation of the matrix, which can be generally written as follows:

(8) $$\frac{d}{d\varphi }\left[\bar{n},\pm k\varphi \right]=\pm k\left[n,\pm k\varphi \right]\left[\bar{w}\right]=\pm k\left[\bar{w}\right]\left[n,\pm k\varphi \right]$$

where the angular velocity matrix, $\bar{w}$, has the following form:

(9) $$\begin{array}{ccc} \left[\bar{w}\right] & = & \left[\begin{array}{ccc} 0 & n_{3} & -n_{2}\\ -n_{3} & 0 & n_{1}\\ n_{2} & -n_{1} & 0 \end{array}\right] \end{array}$$

And its elements were introduced in the calculation program, analogously to the rotation matrix (4), in the following form:

(10) $$w_{ij}=\varepsilon_{ijk}n_{k}$$

Relation (8) can be derived by differentiating the identity matrix:

(11) $$\begin{array}{cccc} \left[E\right] & = & \left[\bar{n},\phi \right] & {\left[\bar{n},\phi \right]}^{T} \end{array}$$

The above relationship is essential for determining the envelope condition of a surface family of the hob. If the envelope condition is written as a mixed product of three vectors, as described by Trajdos [41], the common rotation matrix acting on each vector can be omitted from this product. To do this, the third product vector should be multiplied by an identity matrix constructed based on the common rotation matrix for a given product.

The mathematical analysis of the hob was divided into three parts: a numerical model of the rake surface, flank surfaces, and a cutting edge (rack profile). Starting from the description of the points located on the flank and rake surfaces in the hob coordinate system, the transition to the tool coordinate system was made in successive stages, taking into account the machining kinematics by finding the points common to both surfaces, i.e., the profile of the hob that forms the gear [24,38,42]. Markowski and Rybak postulated the unification of the geometric notation of hob modifications [17]. This was further expanded in the work of Lechowski [35] and especially Nieszporek [37,38,42] and Piotrowski [24]. Special symbols have been introduced in the formulae to ensure the readability and unambiguity of the author’s hob mathematical model described in the article. All symbols with the “p” parameter under the coefficient mean the flank face, while the “n” parameter is assigned to the rake face. The “k” parameter determines the cutting edge (rack profile).

## 3. Geometric Analysis of the Rake Surface

**Figure 3:**
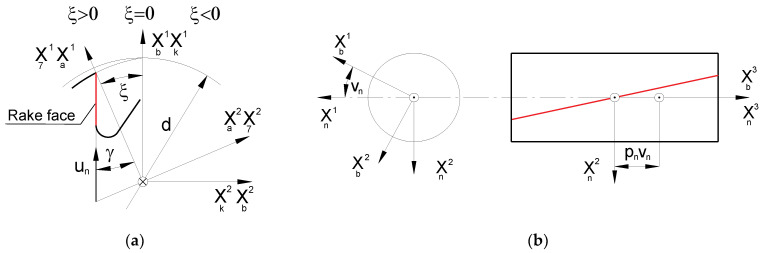
Description of the relative helical movement of the grinding wheel and the hob during the shaping of the rake face: (**a**) profile of the rake face and (**b**) helical movement of the straight profile of the rake face in the hob coordinate system [24].

According to the recommendations of cutting tool manufacturers [2,3,4,10,20,21,22] and the requirements of standards [7,8], the rake face of modern hobs is shaped as a rectilinear helical surface (Figure 3). If the rake angle is zero, this is the Archimedean helical surface, i.e., the surface described by a straight line in helical motion, which has been described by many authors [24,25,26,27,30,38,42]. Depending on the size of the pitch module and the type of material, single-sided conical disc grinding wheels are used to shape the rake face of the hob. Due to the accuracy and difficult-to-machine material (e.g., cemented carbide, innovative materials with very high hardness, exceeding 65 HRC), grinding wheels with a metal body with diamond or borazon abrasive inserts are the most commonly used by hob manufacturers [2,3,4,10,20,21,22].

A rectangular rake face is created by the helical movement of a straight profile (Figure 3) and can be described by the following equation [16,24,27]:

(12) $$\overset{\_}{\underset{n}{r}}=\left[\begin{array}{cc} 3, & v_{n}+\xi \end{array}\right]{\left[\begin{array}{ccc} u_{n}\cos\gamma , & -\frac{d}{2}tg\gamma +u_{n}\sin\gamma , & 0 \end{array}\right]}^{T}+{\left[\begin{array}{ccc} 0, & 0, & p_{n}v_{n} \end{array}\right]}^{T}$$

where u_n_—parameter of the straight profile of the rake face; v_n_—parameter of the helical movement of a straight line; *ξ*—cutter wear angle; *γ*—cutter rake angle; d—external diameter of the hob; and p_n_—rake face parameter (reduced helical surface pitch).

In the production process, the rake face of the hob, which is formed with a single-sided conical grinding wheel, is, in fact, the envelope of the grinding wheel family. A rectangular contour of the rake face is obtained by giving a curvilinear profile of the grinding wheel in its axial cross-section (an arc with a very large radius). This complicates the technological process because it requires, firstly, mathematical calculations to determine the shape of the grinding wheel and, secondly, sharpening the grinding wheel along the appropriate curve determined by the calculations. The correct profile of the grinding wheel was achieved by sharpening it with a single-point diamond sharpener using templates. Currently, modern numerically controlled diamond sharpeners are used instead of traditional templates, which are described by Yoshimo and Ohishima [43], Shuying and Weifang [44], and Nieszporek and Piotrowski [24,37,45]. This allows for virtually any optimisation of the grinding wheel profile. A program controlling the sharpening diamond movement in ISO G-code for CNC machines can be generated directly from the calculation program.

**Figure 4:**
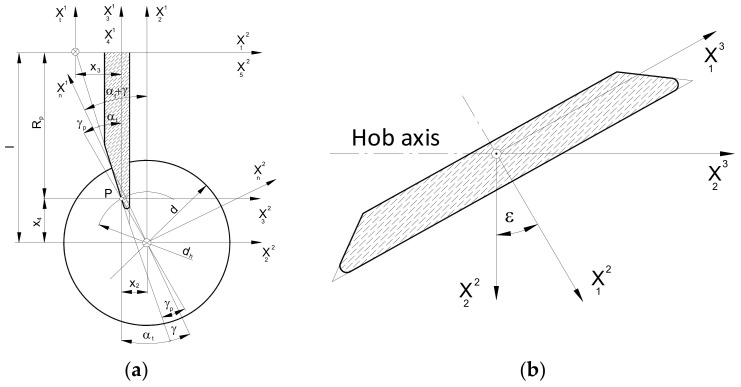
(**a**) Setting of the grinding wheel when shaping the rake face of the hob. (**b**) Angular setting of the grinding wheel in relation to the hob axis [24].

The surface of the grinding wheel is defined as the envelope of the family of rectangular helical rake faces of the hob. The axial profile of the grinding wheel is then determined. During the process of shaping the rake face, the grinding wheel undergoes two movements: a rotational movement relative to its axis (Figure 4a) and a relative helical movement around the hob axis (Figure 4b). However, it is not possible to take both movements into account when transitioning from a hob coordinate system to a grinding wheel coordinate system. Considering the relative helical kinematics in the mathematical notation can lead to identity-fulfilled dependencies, resulting in an error where the iteration process does not converge. This occurs when the helical surface turns into itself during helical motion. For this reason, the relative helical motion of the grinding wheel and the hob has been neglected in the numerical model. Only the relative circular motion of the rake face around the grinding wheel axis was considered (when recording the family of rake faces in the grinding wheel coordinate system; Figure 4).

The given helix angle, *χ*, on the rake face corresponds to a cylinder with a diameter on which this line lies:

(13) $$d_{h}=\frac{Htg\chi }{\pi }$$

where H—the pitch of the helical rake face of the hob; and *χ*—the angle of the helix on the rake face.

This line intersects the frontal profile of the hob rake face at the calculation point, P (Figure 4a). The rake angle of the hob at the calculation point is described as follows:

(14) $$\sin\gamma_{p}=\frac{d}{d_{h}}\sin\gamma$$

where d_h_—the hob diameter for calculating the rake angle of the hob; and *γ*_p_—the rake angle at the given diameter d_h_ (Figure 4a)

As suggested by the authors [24,37,42], the rake-face Equation (1) becomes more complex for the cone-derived rake face. To describe it, a system of two equations should be used. The first is the family equation of the grinding wheel surface in the hob coordinate system.

(15) $$\begin{array}{l} \underset{n}{\bar{r}}=\left[\begin{array}{cc} 3, & -v_{n}+\xi \end{array}\right]\left[\begin{array}{cc} 3, & -\left(\alpha_{nf}+\gamma \right) \end{array}\right]\cdot \\ \left(\begin{array}{l} \left[\begin{array}{cc} 1\;, & -\varepsilon \end{array}\right]\left(\underset{s}{\bar{r}}+{\left[\begin{array}{ccc} r_{n}-\frac{d}{2}\frac{\cos\alpha_{n}}{\cos\gamma }, & -r_{n}tg\alpha_{n}+\frac{d}{2}\frac{\sin\alpha_{n}}{\cos\gamma }, & 0 \end{array}\right]}^{T}\right)+\\ +{\left[\begin{array}{ccc} \frac{d}{2}\left(\frac{\cos\alpha_{nf}}{\cos\gamma }-tg\gamma \sin\left(\alpha_{nf}+\gamma \right)\right), & \frac{d}{2}\left(-\frac{\sin\alpha_{nf}}{\cos\gamma }-tg\gamma \cos\left(\alpha_{nf}+\gamma \right)\right), & 0 \end{array}\right]}^{T} \end{array}\right)\\ +{\left[\begin{array}{ccc} 0, & 0, & p_{n}v_{n} \end{array}\right]}^{T} \end{array}$$

The second one is the envelope condition, which can be written generally as a mixed product of three vectors:

(16) $$f_{n}=\frac{\partial \underset{n}{\bar{r}}}{\partial v_{n}}\frac{\partial \underset{n}{\bar{r}}}{\partial u_{n}}\frac{\partial \underset{n}{\bar{r}}}{\partial \phi_{n}}$$

After taking into account the relationships describing the component vectors, the second component equation describing the envelope condition was written:

(17) $$f_{n}=\left\{\begin{array}{l} {\left[\begin{array}{cc} 1, & -\varepsilon \end{array}\right]}^{T}{\left[\begin{array}{cc} 3, & -\left(\alpha_{nf}+\gamma \right) \end{array}\right]}^{T}\\ \left\{\begin{array}{l} -\left[3\right]\left[\begin{array}{cc} 3, & -\left(\alpha_{nf}+\gamma \right) \end{array}\right]\\ \left(\begin{array}{l} \left[\begin{array}{cc} 1, & -\varepsilon \end{array}\right]\left(\underset{s}{\bar{r}}+{\left[\begin{array}{ccc} r_{n}-\frac{d}{2}\frac{\cos\alpha_{n}}{\cos\gamma }, & -r_{n}tg\alpha_{n}+\frac{d}{2}\frac{\sin\alpha_{n}}{\cos\gamma }, & 0 \end{array}\right]}^{T}\right)\\ +{\left[\begin{array}{ccc} \frac{d}{2}\left(\frac{\cos\alpha_{nf}}{\cos\gamma }-tg\gamma \sin\left(\alpha_{nf}+\gamma \right)\right), & \frac{d}{2}\left(-\frac{\sin\alpha_{nf}}{\cos\gamma }-tg\gamma \cos\left(\alpha_{nf}+\gamma \right)\right), & 0 \end{array}\right]}^{T} \end{array}\right)\\ +{\left[\begin{array}{cc} 3, & -v_{n}+\xi \end{array}\right]}^{T}{\left[\begin{array}{ccc} 0, & 0, & p_{n} \end{array}\right]}^{T} \end{array}\right\} \end{array}\right\}\bar{N}$$

**Figure 5:**
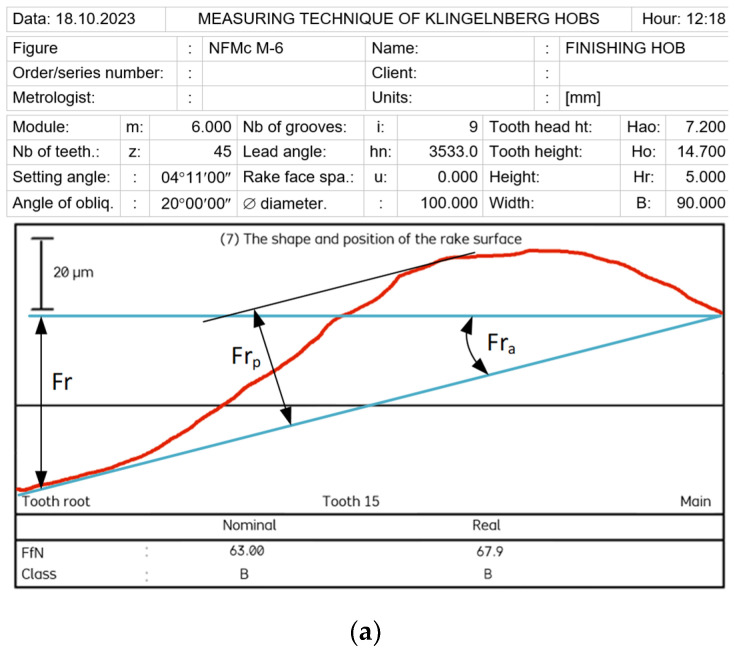

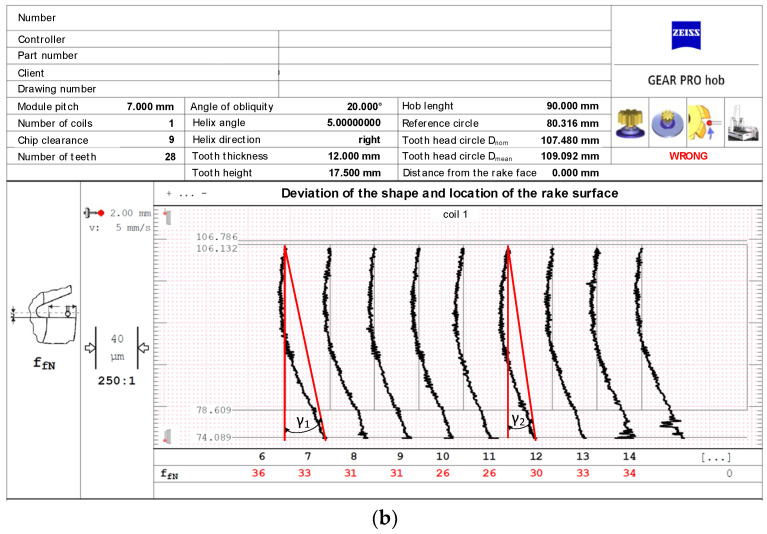
Graphs of straightness and angle error of the rake face. (**a**) Division of the total error (Fr) into angle error (Fr_a_) and straightness error (Fr_p_)—Klingenberg. (**b**) Variation in the rake angle of individual teeth—Zeiss Gear Pro Hob.

The authors’ mathematical analysis revealed that, despite the manufacturers’ recommendations [2,3,4,10,20,21,22] and contrary to the standards [7,8], the rake face of the hob could also be shaped with a single-sided conical grinding wheel with a rectilinear profile in its axial cross-section. This simplifies the technology of shaping the rake face and operating the hob in industrial conditions. The grinding wheel is sharpened in a straight line, reducing costs and facilitating and speeding up the shaping process. As a result of machining, a curvilinear (convex) profile of the rake surface is created, as shown in Figure 5b. However, the analysis of equations, numerical calculations, and measurement results on specialised measuring machines for hobs—Klingerberg [46] and Zeiss Contura CMM [47]—with Zeiss Gear Pro Hob software [48,49] (Figure 5) clearly indicates that the rake face profile is more influenced by rake angle errors (*γ*), resulting from the geometry of the grinding wheel and the relative positioning of the grinding wheel and the hob, rather than the theoretically incorrect curvilinear profile. The rake face profile straightness graph of a user-regenerated class B hob, created with Zeiss Gear Pro Hob software (Figure 5b) [49], revealed that an improperly set grinding wheel caused a deviation in the rake angle. This error was significantly greater than the deviation from straightness. Furthermore, this error caused a variation in the rake angle of individual teeth (*γ*_1_ ≠ *γ*_2_) (Figure 5b) despite the fact that the standard requires this angle to be the same for all teeth.

**Figure 6:**
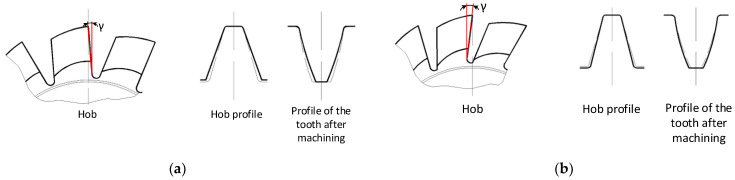
The influence of rake face errors on the profile of the hob and the machined gear: (**a**) rake angle too small and (**b**) rake angle too large [24].

Therefore, according to Figure 5a, the total rake face error Fr is proposed to be divided into the rake angle error Fr_a_ and the rake face straightness error Fr_p_. Limiting the profile optimisation possibilities to a straight rake face restricts the ability to improve the modular hob’s accuracy. Moreover, the inclusion of the tolerance for straightness and radial position of the rake face in the standards [7,8] suggests that it is a zero-rake angle of the hob. Breaking down this deviation into straightness and rake angle errors will apply to hobs with different rake angles. Tolerance of the rake angle is even more important because the impact of rake angle errors on changes in the cutting-edge profile and, thus, on the accuracy of hob cutters is well-known—Figure 6 [15,24].

**Figure 7:**
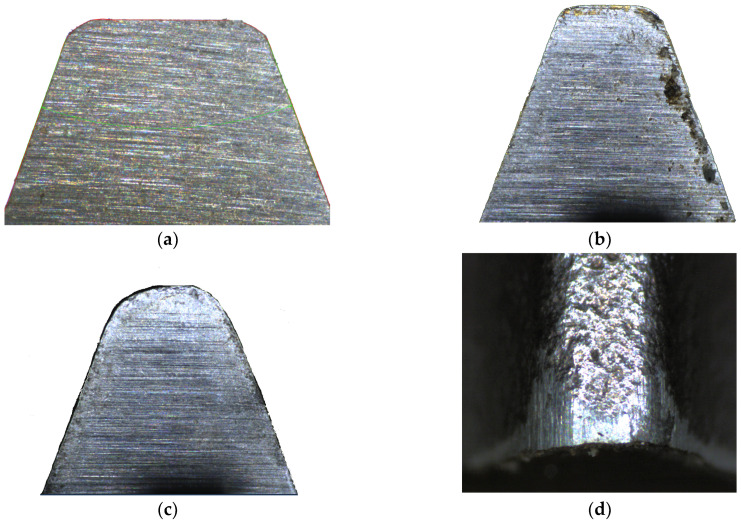
(**a**) Correct tooth profile of the hob. (**b**) Rake face of the hob with the correct profile of the tooth head and visible pits. (**c**) Distorted (rounded) tooth head of the hob due to excessive use—rake face. (**d**) Traces of excessive wear on the hob circumference, tooth head, and flank surfaces.

Another important issue is the sharpening of the hob during its exploitation. The heads of the teeth of the hob are the most heavily loaded during the machining of a gear wheel (Figure 7a). They start milling by cutting into the material first and perform this function throughout the processing time. As a result, the most significant wear occurs on the tooth heads (Figure 7c) and the side edges of the rack teeth (Figure 7b). If the tool is used excessively, wear will occur on the circumference and flank surfaces (Figure 7d), effectively disqualifying the hob from further use. Another problem is chips flowing down the rake face, causing characteristic pits (Figure 7b). A properly used tool must, therefore, be sharpened. For economic reasons, this is very often performed in the toolroom of the hob user. Only the rake face is sharpened using theoretically universal, single-sided disc grinding wheels. This is a cardinal mistake. Yoshino and Ohishima [43] briefly described this, but Nieszporek and Piotrowski [24,38] provided a more detailed explanation. Numerical modelling and measurements have enabled the identification of errors made by users during hob sharpening. As a result, the misshapen profile of the rake profile was transferred to the machined gear. The results confirmed the authors’ numerical model.

Measurements performed using a Garant MM1-300 digital microscope [50,51], CMM machine [47,48,49] (Figure 5b), and special measurement machine for hobs [46] (Figure 5a), together with numerical calculations, have shown that the use of universal curved abrasives leads to significant errors. The profile of the rake face is no longer straight; the rake angle changes; and if the grinding wheel is incorrectly positioned in the chip groove, there is also an error in the rake angle on individual teeth of the rack (Figure 5b). This is due to the fact that the hob manufacturer does not provide any information about the shape of the grinding wheel profile used to shape the rake face finally. Despite the bell-shaped rake face, sharpening with a straight profile wheel results in much more minor errors and should be used in practice.

## 4. Geometric Analysis of the Flank Face

The two flank faces (left and right) of the hob are machined in the turning process using three types of grinding wheels. The most popular are disc-type grinding wheels, which are suitable for most pitch modules [3,20,21,22,30] and do not require any change in the technology used in the rake face machining process. It should be noted that the problem is that the next gear limits the grinding wheel’s run. For large modules (m > 5 (mm)), manufacturers use finger-type grinding wheels [20,21,22,26,36]. The third type of grinding wheel is a pot-type grinding wheel, which requires specialised equipment. For this reason, they are less commonly used by cutting tool manufacturers. Regardless of the grinding wheel and material selected for the hob, the process of shaping the flank faces is technologically complicated. The problem is the small diameter of the disc-type grinding wheel and the low cutting speed, resulting in low machining efficiency and intensive wear of the grinding wheels. This especially applies to tools made of super-hard and innovative materials. This practically eliminates the possibility of using traditional, cheap ceramic corundum (A) or carborundum (SiC) grinding wheels and requires the use of metal grinding wheels with diamond (D) or borazon (BN) abrasives, as in the case of the rake face.

**Figure 8:**
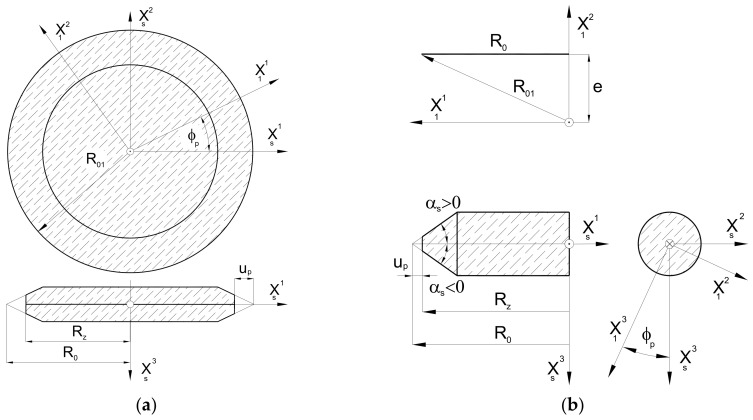
Geometric description of (**a**) disc-type grinding wheel and (**b**) finger-type grinding wheel.

The mathematical model developed by the authors is universal and allows for the use of all three types of grinding wheels (Figure 8). However, due to the scope and complexity of the subject, this article presents an analysis only of the disc-type (Figure 8a) and finger-type (Figure 8b) grinding wheels, which have practically the same profile in axial cross-section. A mathematical model was constructed, considering the method of describing the flat (plane) profile of the grinding wheel and then the setting and kinematics of the grinding wheel movement in relation to the hob. In the generalised form, the plane profile does not coincide with the axial plane of the grinding wheel:

(18) $$\underset{1}{\bar{r}}={\left[\begin{array}{ccc} R_{0}-u_{p}, & e, & u_{p}\cdot tg\alpha_{s} \end{array}\right]}^{T}$$

where u_p_—grinding wheel profile parameter; e—distance of the plane profile from the X^1^ axis of the grinding wheel profile coordinate system; R_0_—parameter of the position of the profile vertex in the plane profile; and α_s_—grinding wheel profile angle.

According to Figure 8, the grinding wheel’s profile can be described in the simplest case by a rectilinear section or, in a generalised form, by a mathematical formula. The authors recommend that it should be a concave or convex arc. In the case of a disc-type grinding wheel, the profile is rotated around the X^3^ axis. In the case of a finger-type grinding wheel, the same profile is rotated around the X^2^ axis, and then the profile reference coordinate system is rotated by an angle π around the X^3^ axis. As recommended by Piotrowski [24] and Nieszporek [38], the surfaces of grinding wheels can be described using a generalised equation:

(19) $$\underset{s}{\bar{r}}=\left[\begin{array}{cc} k_{2}, & -\phi_{p} \end{array}\right]\left[\begin{array}{cc} 3, & k_{1}\pi \end{array}\right]\underset{1}{\bar{r}}$$

where we have

(20) $$k_{1}=\left\{\begin{array}{l} \begin{array}{cc} 0 & \mathrm{disc}-\mathrm{type} \end{array}\;\mathrm{grinding}\;\mathrm{wheel}\\ \begin{array}{cc} 1 & \mathrm{finger}-\mathrm{type}\;\mathrm{grinding} \end{array}\;\mathrm{wheel} \end{array}\right.$$

(21) $$k_{2}=\left\{\begin{array}{l} \begin{array}{cc} 3 & \mathrm{disc}-\mathrm{type}\;\mathrm{grinding}\;\mathrm{wheel} \end{array}\\ \begin{array}{cc} 1 & \mathrm{finger}-\mathrm{type}\;\mathrm{grinding}\;\mathrm{wheel} \end{array} \end{array}\right.$$

**Figure 9:**
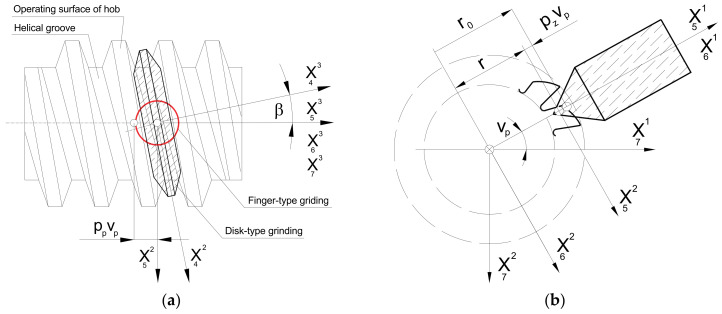
The setting of the (**a**) disc-type grinding wheel in the screw helical groove and (**b**) finger-type grinding wheel with the described staggering motion.

The next step was to describe the setting and the kinematics of the movement of the grinding wheel. To achieve this, the X_S_ coordinate system (as shown in Figure 8) is rigidly connected to the grinding wheel, which describes the surface of the wheel (19). It is now possible to define the relative positioning of the grinding wheel and hob, regardless of the type of grinding wheel selected by the manufacturer. It is important to note that, during the shaping of the flank face, the grinding wheel is placed in a groove located between the adjacent teeth of the rack that defines the profile of the hob. In the case of a disc-type grinding wheel, this means that its rotation at an angle, β, of the helix of the grooves (Figure 9a). This phenomenon does not occur with a finger-type grinder (Figure 9b). However, this parameter is retained to ensure the universality of the mathematical model.

**Figure 10:**
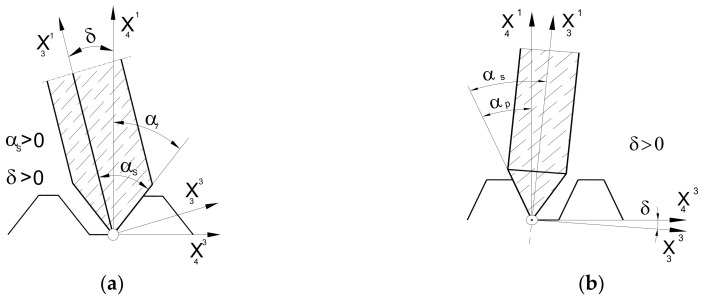
Location of grinding wheels in the helix groove: (**a**) disc-type grinding wheel and (**b**) finger-type grinding wheel [24].

The grinding wheel setting and movement kinematics are described as follows:

1. A transition was made from the X_S_ grinding wheel coordinate system to the auxiliary X_3_ coordinate system related to the grinding wheel vertex point. This was achieved through translation by a radius, R_0_, along the X^1^ axis. (Figure 9);
2. A parameter, δ (angle of asymmetry of the grinding wheel profile), was introduced, describing the tilt of the grinding wheel for the case when the angle of the grinding wheel profile is different from the angle of the hob tooth profile. This parameter is only applicable to disc-type grinding wheels where the left and right sides are shaped at various angles (Figure 10a). For a finger-type grinding wheel, this is not possible (symmetry of the tool in the axial section) and requires the use of two different grinding wheels, with varying angles of conical surface convergence, for the right and left flank faces (Figure 10b);
3. The rotation of the disc-type grinding wheel by the angle β of the coil of the hob’s helix was taken into account around the rotation axis, X^1^, perpendicular to the axis of the grinding wheel and the hob (Figure 9a);
4. The classic solid modular hob is a staggered tool (Figure 1). Therefore, it was necessary to take into account the fact that shaping the side surfaces of the cutter’s teeth is a combination of the rolling and helical motion of the grinding wheel relative to the hob. The staggering process involves the radial back-and-forth movement of the grinding wheel relative to the hob’s rotation axis, every single blade during the hob’s rotation (Figure 9b).

In a generalised form, the equation of the family of surfaces (action surfaces) of the grinding wheel in the hob coordinate system was written as follows:

(22) $$\underset{p}{\bar{r}}=\underset{p}{\bar{r}}\left(\begin{array}{ccc} u_{p}, & \phi_{p}, & v_{p} \end{array}\right)$$

To determine the hob tooth flank face, Piotrowski [24], Nieszporek [38], and Koć [28,29,39] proposed modifying the equation of the surface family (11) by adding the envelope condition in the form of a universal mixed product of three vectors, as described by Trajdos [41]:

(23) $$\underset{p}{f}=\frac{\partial \underset{p}{\bar{r}}}{\partial u_{p}}\frac{\partial \underset{p}{\bar{r}}}{\partial \phi_{p}}\frac{\partial \underset{p}{\bar{r}}}{\partial v_{p}}=0$$

The equation for the grinding wheel flank family, which is the envelope of the grinding wheel family, is written in a universal form with a system of equations. This considers the transformations from the tool (grinding wheel) profile coordinate system to the flank coordinate system, as well as the kinematics and setting of the grinding wheel.

(24) $$\underset{p}{\bar{r}}=\left[\begin{array}{cc} 3, & v_{p} \end{array}\right]\left\{\begin{array}{l} \left[\begin{array}{cc} 1\;, & -\beta \end{array}\right]\left[\begin{array}{cc} 2\;, & -\delta \end{array}\right]\left(\underset{s}{\bar{r}}+{\left[\begin{array}{ccc} R_{01}, & 0 & 0 \end{array}\right]}^{T}\right)\\ +{\left[\begin{array}{ccc} \left(R_{z}-R_{01}\right)\cos\delta +r_{0}-p_{z}v_{p}, & 0, & 0 \end{array}\right]}^{T} \end{array}\right\}+{\left[\begin{array}{ccc} 0, & 0, & -p_{p}v_{p} \end{array}\right]}^{T}$$

(25) $$\begin{array}{l} \underset{p}{f}=\left\{{\left[\begin{array}{cc} 2, & -\delta \end{array}\right]}^{T}{\left[\begin{array}{cc} 1, & -\beta \end{array}\right]}^{T}\left\{\begin{array}{l} \left[3\right]\left\{\begin{array}{l} \left[\begin{array}{cc} 1, & -\beta \end{array}\right]\left[\begin{array}{cc} 2, & -\delta \end{array}\right]\left(\underset{s}{\bar{r}}+{\left[\begin{array}{ccc} R_{01}, & 0, & 0 \end{array}\right]}^{T}\right)\\ +{\left[\begin{array}{ccc} \left(R_{z}-R_{01}\right)\cos\delta +r_{0}-p_{z}v_{p}, & 0 & 0 \end{array}\right]}^{T} \end{array}\right\}\\ +{\left[\begin{array}{ccc} -p_{z}, & 0, & 0 \end{array}\right]}^{T}+{\left[\begin{array}{ccc} 0, & 0, & -p_{p} \end{array}\right]}^{T} \end{array}\right\}\right\}\underset{s}{\bar{n}} \end{array}$$

During the production process, the finger-type grinding wheel enters the inter-tooth helical groove with a certain clearance. This is because two grinding wheels set at different angles are used to shape the left and right flank faces. Therefore, the latest version of the mathematical model includes the option to individually set the amount of clearance, which is equivalent to moving the grinding wheel away from the cutter axis in the radial direction by a certain amount:

(26) $$\Delta r_{op}=\frac{\Delta d_{p}}{2tg\alpha_{s}}$$

**Figure 11:**
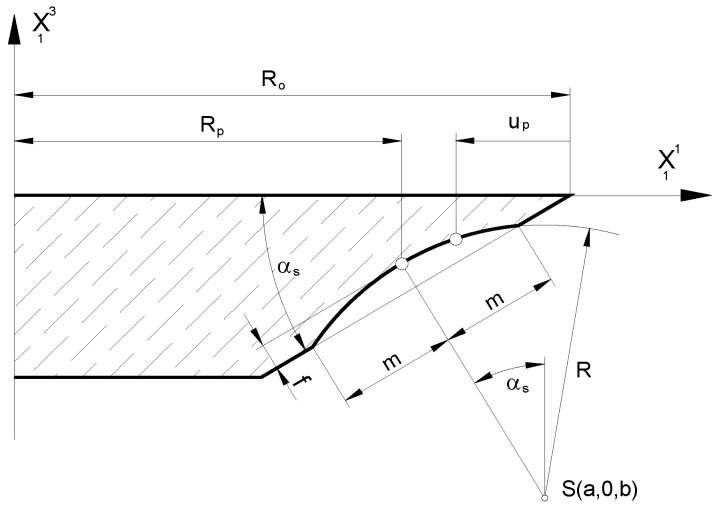
The concave profile of a disc-type grinding wheel—left side.

**Figure 12:**
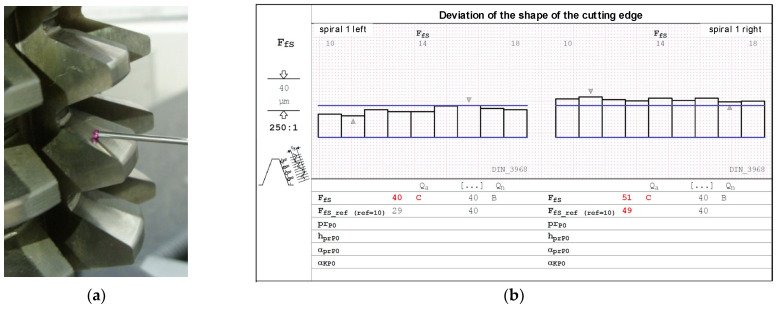
(**a**) Measurement of the flank face profile on the CMM. (**b**) Results of the measurement of the deviation of the shape of the cutting-edge profile.

Adopting the profile of grinding wheels to create a rectilinear flank face is a generally accepted industrial practice. It has been described in detail many times by authors such as Lewkowicz, Lechowski [36], Nieszporek and Legutko [37], Lewkowicz [26], Macioszczyk [27], and Piotrowski [42,52]. However, the results of computer simulations based on the developed mathematical model showed that the grinding wheel’s profile can be shaped along a curve, which increases the accuracy of the hobs. The literature mentions cases where the profile of the grinding wheel is elliptical, postulated by Piotrowski and Nieszporek [24,38], and special cutters are used in the manufacture with a very large negative rake angle, *γ* = −30°, as described by Yoshino and Ohishima [43]. However, the most universal (possible to use on any type of hob) method is to use a curve in the form of an arc with a large radius, i.e., sharpening the disc-type grinding wheel in a circle in its axial section (Figure 11). This is used by hob manufacturers [2,3,4] and has been described theoretically in the work of Lechowski [30], Stachurski and Salamon [53], and Macioszczyk [27]. However, it was only with the use of numerical modelling that the theoretical assumptions could be fully verified and that the accuracy of the grinding wheel profile optimisation could be improved, as described by Piotrowski [24,45]. This is very simple in an industrial environment. The calculation results obtained from the computer program are the data source for numerically controlled diamond sharpeners. In the case of older machines, it is possible to create patterns along which the guide finger of the sharpener moves. The mathematical model considers all four instances of circular shaping of the grinding wheel profile. It is presented in a universal form in Figure 12—considerations for a concave profile—on the left side. Calculations are performed similarly for the remaining three types of profiles, as described by Piotrowski [45,52].

The radius of the substitute profile is as follows (Figure 12):

(27) $$R=\frac{m^{2}+f^{2}}{2\left|f\right|}$$

where R—equivalent profile radius; and f—maximum hob profile error.

(28) $$S\left(\begin{array}{ccc} R_{p}+\left(R-f\right)\sin\alpha_{s}, & 0, & -\frac{\pi m}{4}-\left(R-f\right)\cos\alpha_{s} \end{array}\right)$$

(29) $${\underset{1}{x}}^{3}=b+\sqrt{R^{2}-{\left(a-R_{0}+u_{p}\right)}^{2}}$$

As a result of the introduction of the possibility to shape the grinding wheel profile in an arc, the equation of the grinding wheel flank family as the envelope of the grinding wheel family (24) is supplemented by the following relationship:

(30) $$\frac{\partial {\underset{1}{x}}^{3}}{\partial u_{p}}=\frac{a-R_{0}+u_{p}}{\sqrt{R^{2}-{\left(a-R_{0}+u_{p}\right)}^{2}}}$$

The author’s method is universal and allows any modification of the disc-type grinding-wheel profile to be introduced into the mathematical model and computer program.

**Figure 13:**
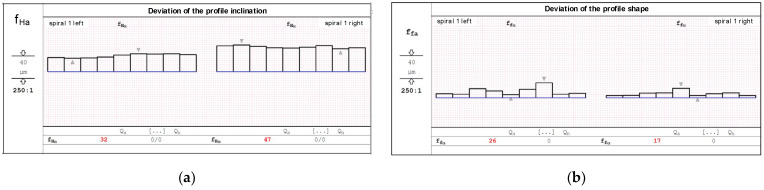
(**a**) Results of measuring the deviation of the profile inclination. (**b**) Results of the measurement of the deviation of the shape of the profile of the cutting shape.

The measurement of a classic hob with module 7, a helical groove angle of 5°, and 5 teeth on a rack was carried out using a CMM [47] and the Zeiss Gear Pro program [48,49]. Before the measurement, the rake face of the hob was sharpened with an incorrectly selected grinding wheel. However, our analysis of the charts (Figure 12 and Figure 13) indicates minimal hob profile errors, consistent with the factory ones. This is because, unlike rake faces, flank faces shaped by the manufacturer do not change over the tool’s life. The users sharpen the tool by reshaping only the rake face. This means that the manufacturer decides on the shape (rectilinear or circular) and accuracy of the flank faces.

## 5. The Profile of the Cutting Edge of a Hob

The cutting edge of a hob is a spatial curve, which is the geometric location of the coordinates of the points that satisfy the flank and rake faces in Equations (15), (17) and (24). The basis for the geometric analysis of hobs, serving as the core of the programme developed by the authors for analysing the profiles of hobs, was, therefore, the numerical determination of the rake and flank faces of the hob and then the numerical determination of the coordinates of the points located simultaneously on both surfaces.

In a generalised form, the equation of the cutting edge (r_k_) of a hob was written by Piotrowski and Nieszporek [24,37,42,52] as follows:

(31) $$\underset{k}{\bar{r}}=\underset{p}{\bar{r}}\left(\begin{array}{ccc} u_{p}, & v_{p}, & \phi_{p} \end{array}\right)\vee \underset{k}{\bar{r}}=\underset{n}{\bar{r}}\left(\begin{array}{ccc} u_{n}, & v_{n}, & \phi_{n} \end{array}\right)$$

There are six parameters in the equations (three for each surface); therefore, to determine the points of the cutting edge, the values of the remaining five parameters had to be determined for the subsequent values of one of the parameters (31). A system of five nonlinear equations relating these parameters was constructed. Two of them are the envelope conditions for the rake face (r_n_) (6) and the flank face (r_p_) (24); the others are obtained from the condition of the intersection of both of these faces (15 and 25) along the cutting edge [24,37,42,52]:

(32) $$\begin{array}{cc} f_{1}=\underset{p}{\bar{r}}\left[1\right]-\underset{n}{\bar{r}}\left[1\right] & \left(a\right)\\ f_{2}=\underset{p}{\bar{r}}\left[2\right]-\underset{n}{\bar{r}}\left[2\right] & \left(b\right)\\ f_{3}=\underset{p}{\bar{r}}\left[3\right]-\underset{n}{\bar{r}}\left[3\right] & \left(c\right)\\ f_{4}=f_{p} & \left(d\right)\\ f_{5}=f_{n} & \left(e\right) \end{array}$$

The formulas in Equation (32) were introduced into the numerical calculation program without writing down the components of the vectors describing the cutter blade surfaces. The equation is fully valid for a conical rake face. However, in the case of a straight rake face (1), there is no envelope condition, so the system of equations is simplified—Equation (32e) becomes redundant. The above system of Equation (32) is solved based on an original, universal calculation program. In the calculation cycle, for given values of one of the parameters, the values of the remaining parameters were determined. After substitution into Equation (31), they allow for the calculation of the coordinates of the points of the cutting edge of the hob blade.

**Figure 14:**
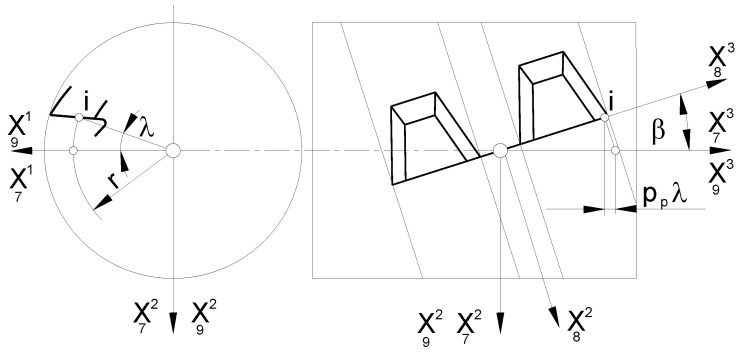
The principle of determining the axial profile of the hob’s operating surface.

To determine the machined surface of the gear wheel as the envelope of the grinding wheel action family, the tool action surface must be determined. The operating face is the geometric location of the cutting edges of the tool’s blades. Two different methods can be used. The first is to move to an infinite number of blades, and the second, which is easier to describe numerically, is to give the cutting edge a helical movement around the hob axis. Once the profile has been defined as a set of points located on it, it is possible to determine the axial profile of the working surface of the hob by transferring these points with a helical movement to the axial plane of the hob (Figure 14).

In this case, presented in the coordinate system (Figure 14), if one of the axes coincides with the axis of the hob and the other axis is perpendicular to the considered plane of the axial profile of the operating face of the hob, one coordinate will be zero (perpendicular to the axial plane). The other coordinate will be equal to the radius of the point cutting edge (equal to the distance of a given point from the axis of rotation):

(33) $${\underset{9}{x}}^{1}=r=\sqrt{{\left({\underset{7}{x}}^{1}\left[i\right]\right)}^{2}+{\left({\underset{7}{x}}^{2}\left[i\right]\right)}^{2}}$$

The third coordinate is equal to the following:

(34) $${\underset{9}{x}}^{3}\left[i\right]={\underset{7}{x}}^{3}\left[i\right]-p_{p}arctg\left(\frac{{\underset{7}{x}}^{2}\left[i\right]}{{\underset{7}{x}}^{1}\left[i\right]}\right)$$

After determining the coordinates of the axial profile points of the operating face of the hob, the profile angle and profile deviations from the straight line were calculated.

## 6. Numerical Simulations of the Accuracy of a Hob Creating the Gears Made of Any Material

**Figure 15:**
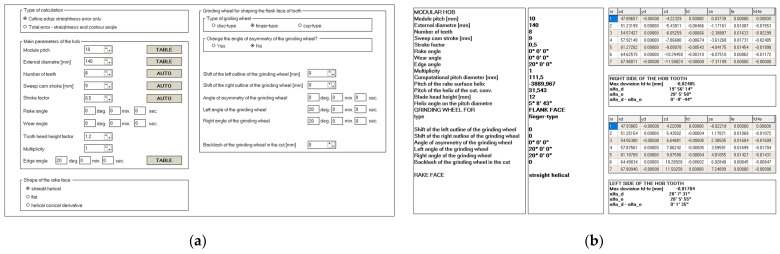
(**a**) Parameters of the hob and machining parameters without optimisation. (**b**) Calculation results of the cutting-edge profile—machining parameters without optimisation.

The analysis presented in the article clearly indicates that the solid modular hob is the most geometrically complex tool used in machining. Developed by the authors and enveloped over the years, the universal mathematical model of the hob makes it possible to improve the accuracy of hobs for various modules and the number of teeth on the rack by changing the shape of the tooth profile of the rack, regardless of the material of the gear wheel and the material used to build the hob. The mathematical model has been used to create a computer system (Figure 15) to optimise the accuracy of the hobs, which directly impacts the accuracy of the machined gear. Tested in cooperation with the Polish machinery industry [20,21,22], it is currently in version 5. The computer program allows for the analysis of the impact of individual parameters on the accuracy of the tools. Initially, the user had to manually modify the input data characterising the hob, the methods of shaping the rake and flank faces, and the geometric data of the grinding wheels. This required repeated calculations using the “trial and error” method. The latest version is equipped with a mechanism that automatically finds the optimum solution for the parameters selected by the user.

Consider the case of a solid hob (Table 1); the side faces of the teeth were shaped with a straight finger wheel without correction (Figure 15).

**Figure 16:**
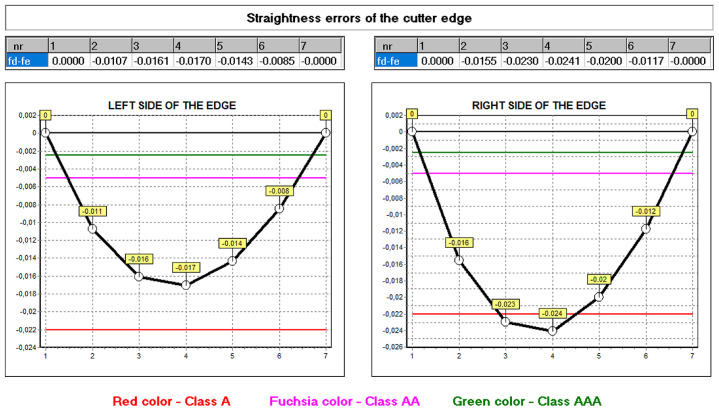
Graph of deviations from the straight profile of the cutting edge—machining parameters without optimisation—hob in Class B.

**Figure 17:**
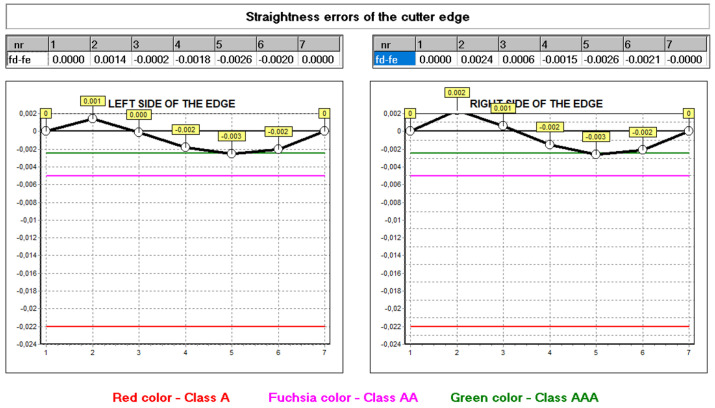
Graph of deviations from the straight profile of the cutting edge—machining parameters without optimisation—hob in Class B.

The hob was classically shaped, in accordance with the standard: the rake face was a rectangular helical surface. The results of calculations and measurements carried out by the authors [45,52,54] showed that the resulting profile errors, in this case, were significant (Figure 16 and Figure 17)—characteristic of Class B tools [7,8].

**Figure 18:**
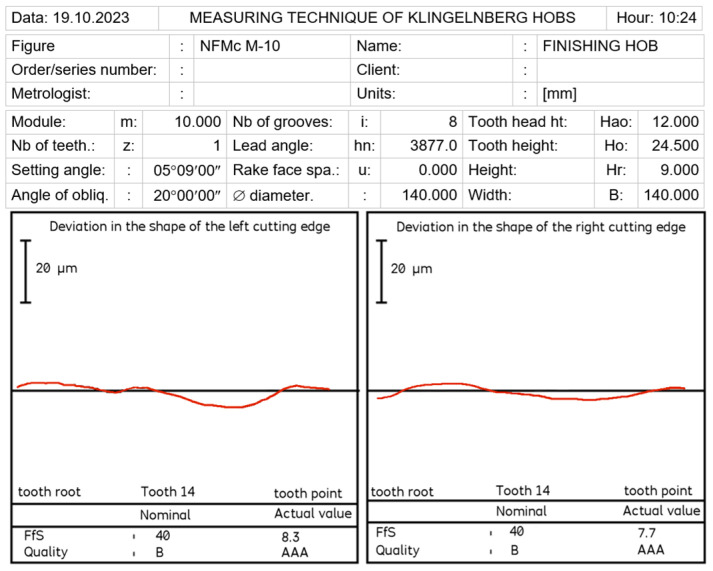
Measurement of deviations from the straight profile of the cutting edge—machining parameters after optimisation, rectangular rake surface hob, module 10, Class AAA.

By carrying out a geometric analysis using proprietary software, it was possible to find an optimum solution that allowed a Class AAA [7,8] tool to be obtained using the same tools (grinding wheels) and machine tools. The angle of the finger-type grinding wheels was modified. The angle for the grinding wheel for shaping the left flank face was 20°15′05″, and for the right one, it was 20°50′00″. It should also be noted that the complex nature of the influence of various factors on the hob profile means that, by changing the angle of the grinding wheel profile, the position of the straight grinding wheel profile must also be corrected. In the presented case, the position (shift) of the left grinding wheel was corrected by 1.2 mm, and the right by 1 mm. As a result of the optimisation, the hob profile is within the parameters of a Class AAA hob (Figure 17) [7,8]. It is characteristic (in the case of hobs whose side faces of the blades are shaped with a finger-type grinding wheel sharpened in a straight line) that, for the optimum solution, the cutter profile (its deviations from the straight line) is concave–convex. This can be considered a criterion for obtaining an optimal solution (assuming that the cutter angle errors are eliminated simultaneously). The measurements of the hob made after modifying the technological parameters confirmed the correctness of the calculations (Figure 18).

**Figure 19:**
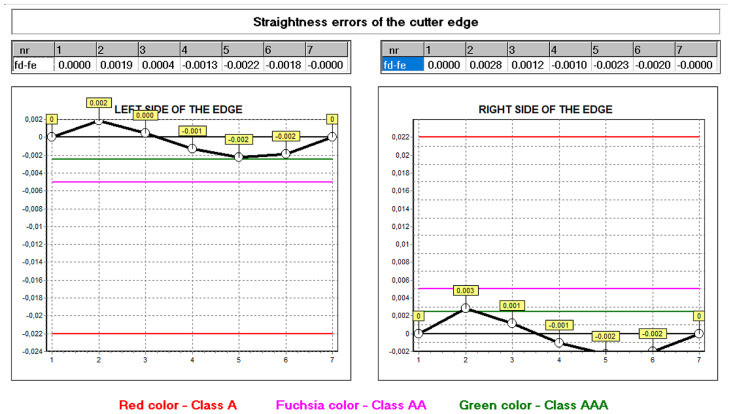
Graph of deviations from the straight profile of the cutting edge—machining parameters after optimisation; conical rake face—hob Class AAA.

The next step was to analyse the use of the conical rake face (Figure 19). The same parameters were used in the calculation as for the straight helical rake face. The rake face was shaped using a grinding wheel with a disc-type and a radius of 70 mm at an angle of 15°. The results of the calculations and measurements confirmed that it is possible to use this type of face, even though the rake face has a characteristic convex shape. The deviations are within the range of Class AAA hobs and differ by only a few micrometres from the results obtained for a rectangular surface (Figure 19). The convex surface will have little effect on the life of the cutting edges. It should not be used for innovative materials for gears that require very sharp cutting edges and a straight-line rake face, particularly polymer materials. However, suppose that the use of a straight or conical hob rake surface does not have a significant impact on the accuracy of the profile. In that case, the latter should be used for technological reasons.

**Figure 20:**
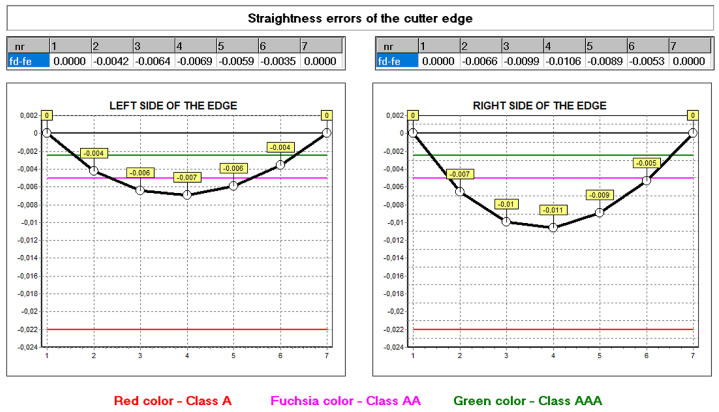
Graph of deviations from the straight profile of the cutting edge for a hob with an increased external diameter of D = 180 mm—machining parameters without optimisation; straight rake face—hob A class.

**Figure 21:**
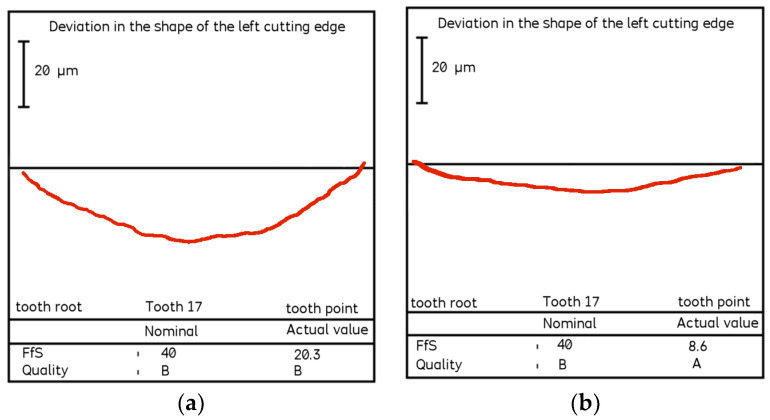
Comparison of the measurement results of the left edge of the hob rack profile: (**a**) hob with a standard diameter of 140 mm and (**b**) hob with an enlarged diameter of 180 mm.

The developed mathematical model and the computer program allow for the analysis of the influence of other construction and technological parameters (input data) on the accuracy of the hob profile. In practice, it was not always possible to take this into account. This was explained in more detail in the works by Kunstetter [9], and Lewkowicz and Lechowski [36]. The authors proposed modifying the hob profile and structure only theoretically, without the possibility of numerically verifying the results of design changes. The first numerical model was proposed by Mazurowicz and Raczyk [40]. However, it was limited to hobs with protuberances. Only the results of numerical simulations and measurements conducted by Nieszporek [37,38] and Piotrowski [24] allow us to conclude that the external diameter of the hob has the most significant influence. Calculations performed for identical technological parameters, except for the external diameter of the hob, which was changed from 140 mm to 180 mm, showed a significant difference in profile accuracy (Figure 20 and Figure 21).

Computer modelling made it possible to identify differences in the shape of the profile and the size of the defects. The comparison of Figure 16 and Figure 20 shows that a tool with a larger diameter (without optimisation) is already in the accuracy range of class A hobs. A 180 mm diameter hob was made to verify the calculation results, and the deviations from a straight line were measured (Figure 21) on a Klingelnberg machine. The measurement results confirmed the correctness of the calculations. The differences in the hob accuracy class result from the fact that, as the screw diameter increases, the helix angle of the screw spiral decreases. If the external diameter of the screw tends to infinity, then in the limit case, we do not shape a screw surface but a groove on the circumference of the cylindrical surface (the angle of the helix of the screw turns is zero). In this case, it is not hobbing machining but shaping machining, during which the axial profile of the grinding wheel is precisely reproduced on the axial profile of the machined surface (the axis of the finger-type or disc-type grinding wheel and the axis of the screw are perpendicular or parallel, and they lie in one plane). For this reason, there are no hob profile errors. It follows that hobs with larger diameters are much more accurate. However, in practice, it is impossible to increase the hob’s diameter without limitations due to space problems for the headstock of the hobbing machine [16,23].

As a result of the above considerations, the interpretation of the influence of multiplicity on the change in the accuracy of the hob’s profile becomes clear. As the multiplicity (convolution) increases, the pitch rises (equal to the product of the axial pitch and the multiplicity), i.e., the helix angle of the hob’s turns, and, therefore, the profile errors of the hob increase. The highest precision hobs have to be made with a multiplicity equal to 1. The calculation and measurement results are different for the tooth’s left and right cutting edges (Figure 18). This is due to the effect of staggering on the lateral clearance angles of the left and right flanks. If we switch off staggering in the calculation process (grind the screw), the deviations for both edges will be the same. On the one hand, there is the effect of the addition of the corresponding angles (the helix angle and the angle resulting from the sweep of the blade) and, on the other hand, their subtraction. As a result, the interpretation of the influence of the number of blades on the circumference of the hob and the sweep cam pitch on the hob’s accuracy is complex and ambiguous.

## 7. Conclusions

Manufacturers of hobs endeavour to create tools in Classes AA to AAAA, which is a consequence of the requirements of the machinery industry, particularly the automotive industry. The analysis indicates that it is impossible to achieve Class AA milling cutters through practical “trial and error” (which was employed by Polish manufacturers of hobs) due to the intricate nature of the influence of construction and technological parameters on the cutter’s outline. Only the developed model made it possible; the mathematical model of a hob developed following extensive research, and it enabled the analysis of the rake and flank faces and their impact on the accuracy of the hob rack profile. The rake surface should be rectangular, and the standards describe only the total error of this surface. According to the calculation results, it is postulated that this error should be divided into the rake angle error and the straightness error. It was also proposed that a conical rake surface be used, which would simplify the technology of hobs and be beneficial for both the manufacturer and the user of these tools. A crucial question arises as to the significance of the profile of the rake surface. It is sufficient to limit oneself to the profile of the cutting edge of the hob. Suppose the profile of the cutting edge lies on the involute helical surface and is, therefore, accurate. In that case, the rake face profile should be considered correct regardless of the accuracy class it is in due to deviations in the straightness and radial position of the rake face profile.

In the case of hobs whose side face of the teeth is shaped with a finger-type grinding wheel, the method of correcting the grinding wheel profile, which involves sharpening it in a straight line at an appropriate angle in a plane parallel to the cutter axis, is an effective means of obtaining Class AA cutters. Different grinding wheels should be used to shape the left- and right-side surfaces of the blade. If the side flanks of the teeth are shaped with a disc grinding wheel, sharpening this grinding wheel in an axial section along a circular arc at an appropriate angle (the angle of the contour, i.e., the direction of the chord of the arc of the axial outline of the grinding wheel) allows us to obtain Class AA milling cutters. The utilisation of both technologies permits the production of cutters of even higher quality, with the potential to achieve Class AAA or AAAA hobs.

Knowledge of the geometry of the hob allows for an analysis of its impact on the accuracy of the gear wheel. The research and computer simulations made it possible to determine which of the parameters—gear material, machining method, or geometric shape of the hob—had the most significant effect on the accuracy of the gear and, consequently, the gear transmission. In conclusion, the mathematical model of the modular solid hob presented in the article and the computer program developed on its basis allow for the optimisation of the profile of the rack of the hob. The most important fact is that, with such a geometrically composed cutting tool and hobbing process, it is not the gear material, even an innovative one, that determines the accuracy of the gear. The most significant influence is the geometry of the hob and, in fact, the shape of the profile of the rack and its correct positioning in relation to the gear being machined on the hobbing milling machine. The other technological parameters—feed, cutting speed, and cutting fluids—have a secondary influence (if they are technologically correct). This was also confirmed in the previously mentioned literature [30,31,32,33,34] and by Timofeev, Sachkov, Koand valevich [55]; and Dazhu, Jiang, Xiaoqing, and Lian [56] in their works on the shaping of gear teeth.

Of course, the material of the gear determines the quality of the cutting process and, indirectly, the quality of the surface after machining. Innovative materials with unusual fillers can cause high roughness of the gear after milling, and such a wheel will need to be ground later. The gear’s material directly affects the life of the tool, especially those with a hardness greater than 65 HRC or with additives that affect the cutting properties, and determines the type of hob used. Composite [10] or monolithic cemented carbide hobs [2,3,4] with extended tooth lengths are used for materials that are difficult to machine. It should be noted that composite hobs are used only for machining gears up to Class 10 [8,15]. This is due to the lower accuracy of the hobs caused by problems with the correct positioning of the sockets’ inserts, as explained by Piotrowski, Boral, and Gołębski [57]. Modern hobs can be used to machine virtually all gears made from machinable materials, including innovative materials used for special applications. The computer program developed by the authors and constantly improved allows us to find the best tool geometry, including the modification of the tooth profile of the gear) and technology—the geometry of the cutter position in relation to the gear to be machined.

## Figures and Tables

**Table 1:** Parameters of the calculated hob.

Parameter	Pitch Module	External Diameter	Number of Teeth	Staggered Stroke	Profile Angle	Multiplicity	Rake Angle
Value	10 mm	140 mm	8	9 mm	20°	1	0°

## Data Availability

The raw data supporting the conclusions of this article will be made available by the authors upon request.
